# CTRP10 is required for optimal motor function

**DOI:** 10.1016/j.jbc.2026.111420

**Published:** 2026-03-31

**Authors:** Fangluo Chen, Muzna Saqib, Chantelle E. Terrillion, Noelle Wright, Dylan C. Sarver, Joseph Scafidi, G. William Wong

**Affiliations:** 1 Department of Physiology, Pharmacology and Therapeutics, Johns Hopkins University, School of Medicine, Baltimore, Maryland, USA; 2 Department of Psychiatry and Behavioral Sciences, Johns Hopkins University, School of Medicine, Baltimore, Maryland, USA; 3 Department of Neurology, Johns Hopkins University, School of Medicine, Baltimore, Maryland, USA; 4 Michael V. Johnston Center for Developmental Neuroscience, Kennedy Krieger Institute, Baltimore, Maryland, USA

**Keywords:** cerebellum, CTRP, mouse behaviors, motor cortex, motor function, mitochondrial respiration

## Abstract

CTRP10, also known as C1QL2, is a secreted protein of the C1q family. In the central nervous system, CTRP10 is predominantly expressed by neurons and oligodendrocytes. Changes in brain expression of *CTRP10* are associated with addiction, depression, and psychiatric disorder. CTRP10 also serves as a specific molecular marker for the excitatory neurons in the anterior thalamic nuclei complex, as well as for neurons that control proprioception and fine motor skills. Whether CTRP10 is required for nervous system control of behaviors is unknown. Here, we determine whether and how CTRP10 deficiency adversely impacts mouse behaviors. Constitutive knockout (KO) mice lacking CTRP10 have normal exploratory behaviors, spatial and recognition memory, and sensorimotor gating. While grip strength is preserved, *Ctrp10* KO female mice show impaired motor coordination in the rotarod task; motor learning, however, is intact. Beam walk and complex running wheel tasks further indicate striking deficits in fine motor skills, with female KO mice showing markedly more pronounced phenotypes. Transcriptomic analyses show that CTRP10 loss alters biological pathways in the cerebellum and motor cortex related to synaptic organization, cell signaling, mitochondrial respiration, and locomotor behavior. Functional analysis also indicates reduced mitochondrial respiration in the motor cortex of KO mice. These combined changes likely contribute to motor function deficits in our KO animals. Collectively, our data uncover a novel central function of CTRP10 and provide genetic evidence that CTRP10 is required for optimal gross and fine motor function.

The highly conserved C1q protein family comprises over thirty members in human, mouse, and other vertebrates (1, 2, 3). These include the immune complement C1q, adiponectin, C1q/TNF-related proteins (CTRP1-15), cerebellins (Cbln1-4), multimerins (Mmrn1-2), Emilins (Emilin1-3), caprin-2, and collagen VIII and X (3, 4). These proteins share a common globular C1q domain in their C-termini (5, 6, 7). With the exception of caprin-2 (8), C1q family members are secreted proteins with a canonical signal peptide, and most circulate in blood (9). They function as either autocrine, paracrine, and/or endocrine factors, and are known to play diverse and pleiotropic roles, ranging from immunity (10), metabolism (11, 12, 13, 14, 15, 16, 17, 18, 19, 20, 21), inflammation (19, 22, 23, 24), renal, gastrointestinal, and cardiovascular physiology (22, 25, 26, 27, 28, 29, 30, 31), tissue repair (32), and visual and central nervous system functions (33, 34, 35, 36, 37, 38, 39).

We originally identified CTRP10 (9) (GenBank accession # AAY21934) on the basis of shared sequence homology to adiponectin and other CTRP members (40). While we have defined the *in vivo* metabolic function for most of the CTRP family members (11, 12, 13, 14, 15, 16, 17, 18, 19, 20, 21), there is limited information regarding the physiologic function of CTRP10 (also known as C1QL2). Using a genetic loss-of-function mouse model, we recently showed that CTRP10 is required for body weight control in female mice and its deficiency uncouples obesity from metabolic dysfunction (41). CTRP10 is also known to play important roles in the central nervous system (CNS). As a transsynaptic organizer secreted from the mossy fibers, Ctrp10/C1ql2 promotes the proper clustering of kainite-type glutamate receptors (GluKs) on the hippocampal CA3 pyramidal neurons; it directly binds to neurexin3 (Nrx3) on the presynaptic terminals and to GluK2 and GluK4 on the postsynaptic terminals (42). In mice lacking both C1ql2 and C1ql3/Ctrp13, the number of seizure events induced by pilocarpine is significantly lower as a consequence of reduced recruitment of postsynaptic GluKs in the dentate gyrus. Additionally, it has been shown that the interaction of Ctrp10/C1ql2 with Nrx3 helps recruit vesicles to the synapse and strengthen synaptic connections in the hippocampal CA3 mossy fibers (43).

Other putative roles for CTRP10 in the CNS have also been suggested. For example, genome-wide association studies have linked *CTRP10*/*C1QL2* to cocaine use disorder (44). In rat models of depression, *CTRP10* expression increases in the dentate gyrus but decreases in the nucleus accumbens (45). In individuals with a history of psychiatric disorders such as schizophrenia, *CTRP10* expression is elevated in the dorsolateral prefrontal cortex of both males and females (45). Recently, CTRP10/C1QL2 was found to be a molecular marker for the excitatory neurons in the anterodorsal thalamus of the anterior thalamic nuclei complex, a brain region involved in cognitive tasks and contextual memory encoding (46). Despite these associations, the direct contribution of CTRP10 to addictive behavior, cognition, and psychiatric disorders remains unknown. Interestingly, *Ctrp10* also serves as a molecular marker for the excitatory rubral cells in the red nucleus that modulate fine motor skills such as reaching and grasping (47). Single-cell RNA sequencing further identified *Ctrp10* as a molecular marker of proprioceptor subtypes that innervate the abdominal muscle of mice (48). Whether CTRP10 has a direct role in fine motor control and proprioception, however, has not been established.

Since pathophysiological states alter *CTRP10* expression in the CNS, we hypothesized that this synaptic protein may have previously unknown roles in regulating behaviors. To determine this, we performed a battery of behavior tests on WT and constitutive *Ctrp10* KO mice. We showed that CTRP10 deficiency does not affect exploratory and anxiety-like behaviors, spatial and recognition memory, and sensorimotor gating in either male or female mice. However, loss of CTRP10 significantly impaired motor skill and coordination in female mice, with male mice exhibiting a milder phenotype. These deficits are not due to intrinsic weaknesses in the skeletal muscle as grip strength is preserved in the *Ctrp10* KO mouse. Instead, we observed major transcriptomic changes in the cerebellum and motor cortex of *Ctrp10* KO mice. The major biological pathways and molecular functions affected by CTRP10 deficiency include those related to synaptic organization, cell signaling, mitochondrial respiration, and locomotor behavior. Functionally, we also observed reduced mitochondrial respiratory activity in the motor cortex. Alterations in the two critical brain regions that control motor function likely contributed to the motor deficit phenotypes seen in *Ctrp10* KO animals. Collectively, our data highlight a novel function of CTRP10 in the brain and its requirement for optimal motor function.

## Results

### Expression of CTRP10 in the central nervous system

**Figure 1:**
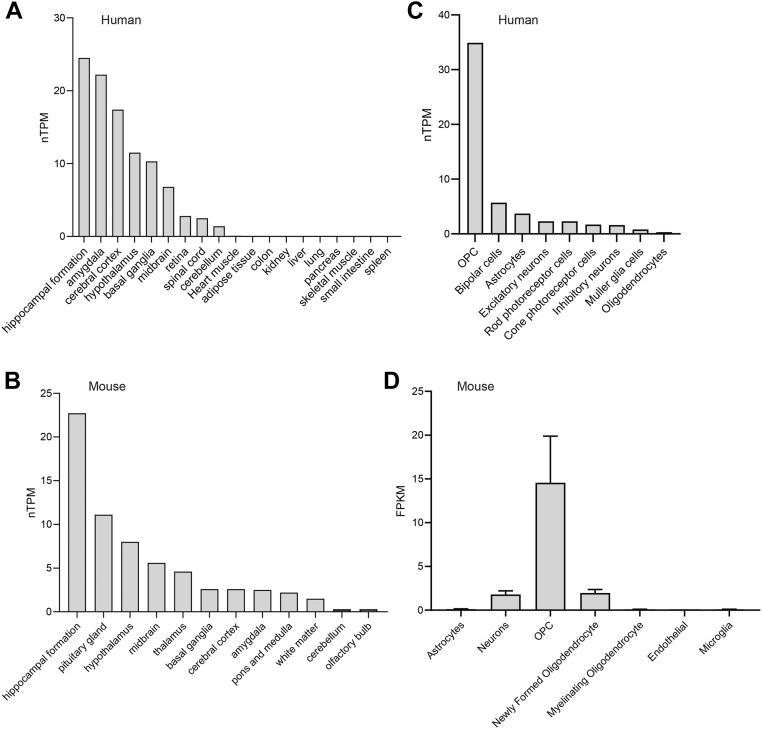
**CTR10 expression in the central nervous system**. *A* and *B*, CTRP10/C1QL2 transcript expression across normal human (*A*) and mouse (*B*) brain regions based on the consensus Human Protein Atlas and Gene-Tissue Expression (GTeX) datasets (49, 50). The data can be accessed *via* the Human Protein Atlas database (proteinatlas.org). nTPM denotes normalized protein-coding transcripts per million and it corresponds to the mean values of the different individual samples from each tissue. *C*, expression of human *C1QL2*/*CTRP10* transcript in different cell types within the brain based on single-cell RNAs-seq data (proteinatlas.org). *D*, expression of mouse *C1ql2*/*Ctrp10* transcript in different cell types within the brain (51). The data can be accessed *via* the Brain RNA-Seq database (brainrnaseq.org).

In humans, the brain has the highest expression of *Ctrp10*/*C1ql2* (Fig. 1*A*) (49, 50). Similarly, in the mouse, *CTRP10*/*C1QL2* transcript is also predominantly expressed in the brain, across the major brain regions (Fig. 1*B*) (50). While neurons express CTRP10, oligodendrocyte progenitor cells have the highest expression of *CTRP10* in mice and humans (Fig. 1, *C* and *D*) (51). However, in a more recent study of adult mouse brain using high-throughput single-cell RNA sequencing technology (Drop-seq), *Ctrp10*/*C1q2* was found to be most highly expressed in the neurons of hippocampus and substantia nigra (52). Given the limited functional information of CTRP10/C1QL2, its expression patterns within the CNS suggest an undiscovered central role of this secreted protein in regulating physiology and behavior.

**Figure 2:**
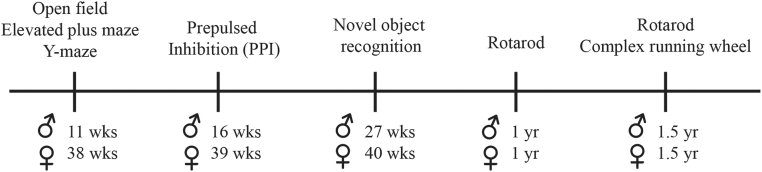
**Timeline of behavioral tests and the age of mice**. A, diagram indicating the chronological order of the behavioral tests and the age of male and female mice at the time of behavioral assessments.

### Loss of CTRP10 does not affect exploratory and anxiety-like behaviors

**Figure 3:**
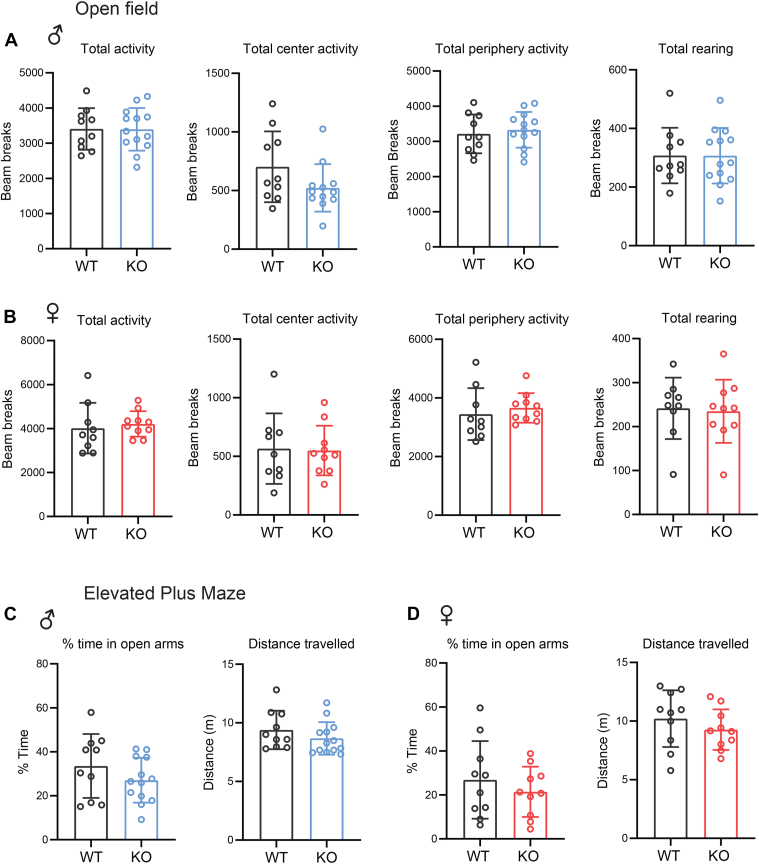
**CTRP10 deficiency does not affect exploratory and anxiety-like behaviors**. *A* and *B*, open field tests of male (*A*) and female (*B*) WT and *Ctrp10* KO mice. The amount of total activity spent at the center and periphery of the open field, as well as rearing activity, were not significantly different between genotypes of either sex. *C* and *D*, elevated plus maze tests of male (*C*) and female (*D*) WT and KO mice. The percent of time spent in the open arms and the total distance traveled were not significantly different between genotypes of either sex. All data are presented as mean ± S.D. Sample size: males (WT = 10; KO = 13) and females (WT = 10; KO = 10).

We carried out a battery of tests to determine if CTRP10 in the CNS is required for modulating mouse behaviors. The timeline of behavioral tests and the age of the male and female mice are indicated (Fig. 2). First, we assessed whether CTRP10 deficiency affects exploratory behaviors. In an open field test for exploratory and anxiety-like behavior, both male and female *Ctrp10* KO mice spent similar amounts of time in the center and periphery of the open field, and had similar numbers of rearing activity, as the WT controls (Fig. 3, *A* and *B*). In the elevated plus maze that measures exploratory and anxiety-like behavior; both male and female *Ctrp10* KO mice also spent a similar proportion of time exploring the open arms as the WT controls (Fig. 3, *C* and *D*). Together, these data indicate that CTRP10 deficiency does not affect general exploratory and anxiety-like behaviors.

### Mice lacking CTRP10 have normal spatial working and recognition memory

**Figure 4:**
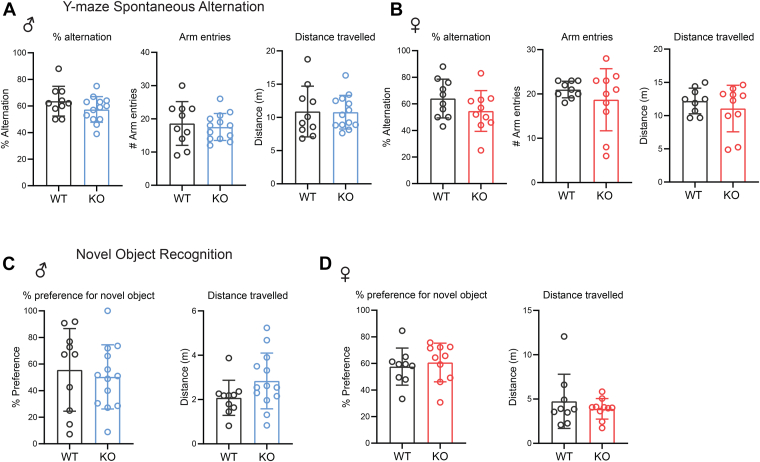
**Loss of CTRP10 does not impair spatial working and recognition memory**. *A* and *B*, Y-maze spontaneous alternation tests of male (*A*) and female (*B*) WT and *Ctrp10* KO mice. The percent alternation, arm entries, and total distance traveled were not significantly different between genotypes of either sex. *C* and *D*, novel object recognition tests of male (*C*) and female (*D*) WT and KO mice. The percent preference for novel object was not significantly different between genotypes of either sex. All data are presented as mean ± S.D. Sample size: males (WT = 10; KO = 13) and females (WT = 10; KO = 10).

Next, we performed behavior tests to assess CTRP10 requirement for spatial working and recognition memory. We used the Y-maze spontaneous alternation test to measure short-term spatial working memory (53). The total distance traveled, number of arm entries, and percent correct alternation between the three arms were similar between WT and *Ctrp10* KO male and female mice (Fig. 4, *A* and *B*). Next, we performed the novel object recognition test, a behavioral test that measures short-term recognition memory (54). In male and female mice lacking CTRP10, the percent preference for the novel object was not significantly different from WT controls (Fig. 4, *C* and *D*). Together, these results indicate that CTRP10 is not required for spatial working and recognition memory.

### CTRP10 deficiency does not affect sensorimotor gating

**Figure 5:**
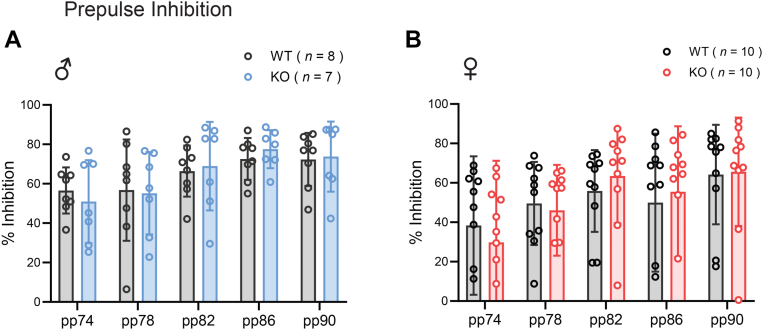
**CTRP10 deficiency does not affect sensorimotor gating**. *A* and *B*, prepulse inhibition of acoustic startle response in male (*A*) and female (*B*) WT and KO mice. Percent of inhibition in acoustic startle response following different prepulse (74–90 dB) were not different between genotypes of either sex. All data are presented as mean ± S.D. Sample size: males (WT = 8; KO = 7) and females (WT = 10; KO = 10).

Since *CTRP10* expression is altered in the brain of individuals with neuropsychiatric disorders (45), we examined whether CTRP10 deficiency affects neuropsychiatric-like behaviors. It is known that humans with neuropsychiatric disorders (*e*.*g*., schizophrenia), as well as mouse models with schizophrenia-like phenotypes, have reduced prepulse inhibition of acoustic startle response (55, 56). In response to different prepulse intensities, *Ctrp10* KO male and female mice showed similar degrees of inhibition to subsequent acoustic startle response as the WT controls (Fig. 5, *A* and *B*). These results indicate that CTRP10 is dispensable for sensorimotor gating.

### Mice lacking CTRP10 have normal grip strength but impaired motor coordination

**Figure 6:**
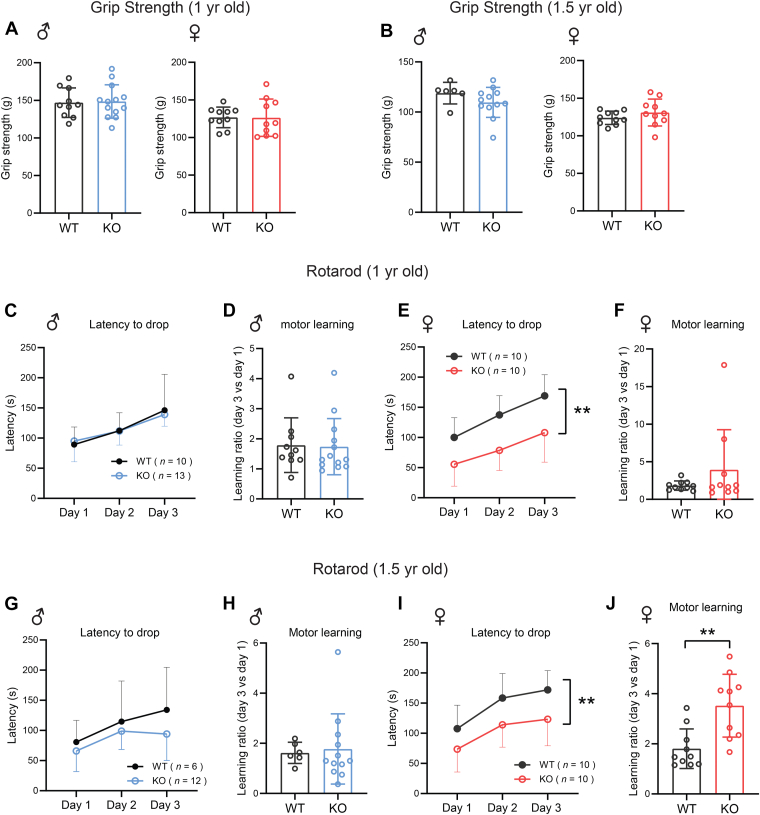
**Loss of CTRP10 impairs motor coordination**. Grip strength analysis of WT and KO male and female mice at 1 year (*A*) and 1.5 years (*B*) of age. Rotarod analysis of motor coordination and motor learning in WT and KO male and female mice at 1 year (*C*–*F*) and 1.5 years (*G*–*J*) of age. Latency to drop from the rotarod over a 3-day period of testing was significantly different between genotypes in female, but not male, mice. Sample size: males (WT = 10 and KO = 13) and females (WT = 10 and KO = 10) at 1 year of age; males (WT = 6 and KO = 12) and females (WT = 10 and KO = 10) at 1.5 years of age. All data are presented as mean ± S.D. For rotarod test, ∗∗*p* < 0.01 (between genotypes; 2-way ANOVA with Geisser-Greenhouse correction). For motor learning, ∗∗*p* < 0.01 (between genotypes; unpaired two-tailed student *t* test).

We first performed a grip strength test to evaluate whether CTRP10 deficiency affects intrinsic skeletal muscle function. Both male and female mice lacking CTRP10 had similar grip strength as the WT controls at 1 year of age (Fig. 6*A*). To rule out age-dependent effects, we reevaluated grip strength in the same cohort of mice at 1.5 years of age. Again, grip strength was not different between genotypes of either sex (Fig. 6*B*). Next, we used a standard accelerating rotarod to assess motor coordination and motor learning (57, 58, 59, 60). In normal WT male and female mice, the latency time to drop from the accelerating rotarod improved with successive trials over a 3-day period, indicative of motor learning (Fig. 6, *C* and *E*). At 1 year of age, *Ctrp10* KO female, but not male, mice showed impaired motor coordination when compared to WT controls, as indicated by the shorter latency time to drop from the accelerating rotarod (Fig. 6, *C* and *E*). Male and female *Ctrp10* KO mice, however, had intact motor learning; their performance on the accelerating rotarod improved to the same extent as the WT controls over a 3-days trial period, as quantified by the ratio of day 3 vs day 2 performance (Fig. 6, *D* and *F*). We also reevaluated motor coordination in the same cohort of mice at 1.5 years of age to determine if the motor phenotype gets worse with age. We again observed impaired motor coordination in female, but not male, KO mice (Fig. 6, *G* and *I*), highlighting the robustness of the phenotype; however, the motor phenotype did not appear to get worse with age. While motor learning was not different between genotypes in males (Fig. 6H), female KO mice showed greater motor learning over a 3-days trial period compared to WT controls (Fig. 6J). Together, these data indicate that CTRP10 is required for optimal gross motor function but not motor learning.

### CTRP10 deficiency impairs motor skill

**Figure 7:**
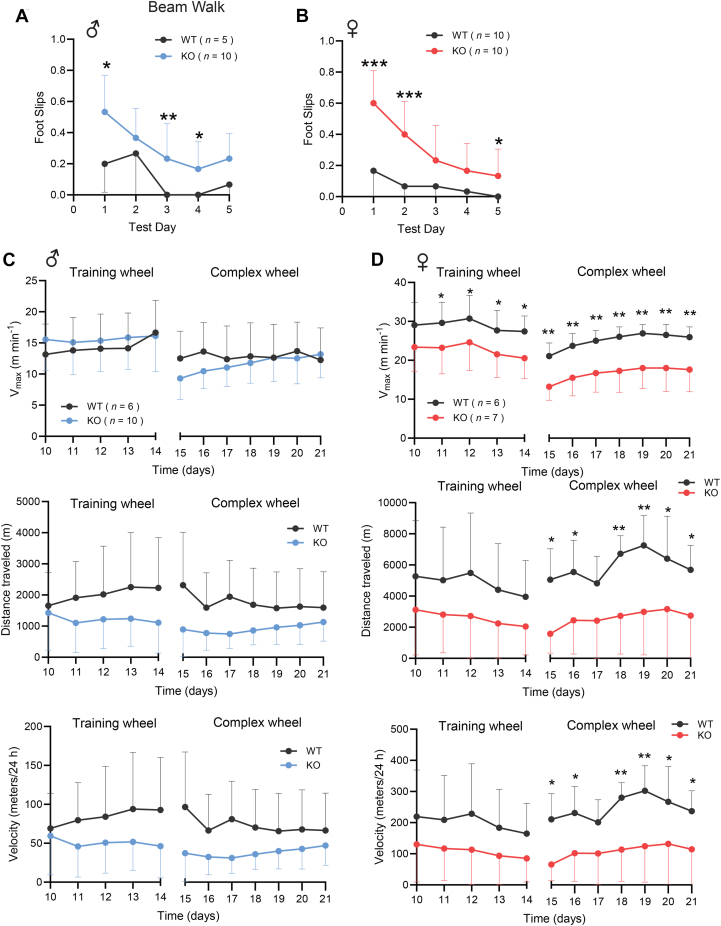
**CTRP10 deficiency impairs motor skill**. Evaluation of motor skill in WT and *Ctrp10* KO male (*A*) and female (*B*) mice at 19 months of age using an inclined beam walk test. Sample size: males (WT = 5 and KO = 10) and females (WT = 10 and KO = 10). *C* and *D*, in the complex running wheel task, male (*C*) and female (*D*) mice were first exposed to a normal/training wheel for 2 weeks, then switched over to a complex running wheel missing 22 rungs in an alternate pattern. Performance parameters—daily maximal speed (Vmax), distance traveled, and average velocity—are collected and quantified. Sample size: males (WT = 6 and KO = 10) and females (WT = 6 and KO = 7). All data are presented as mean ± S.D. All data are presented as mean ± S.D. ∗*p* < 0.05; ∗∗*p* < 0.01; ∗∗∗*p* < 0.001 (2-way ANOVA with multiple comparisons).

Next, we determined whether *Ctrp10* KO mice have a deficit in fine motor skill using the beam walk and complex running wheel tests (61, 62). In the inclined beam walk task, both male and female *Ctrp10* KO mice showed a clear deficit in motor skill as indicated by the higher number of foot slips, with females exhibiting a more pronounced phenotype (Fig. 7, *A* and *B*). However, both WT and *Ctrp10* KO mice of either sex showed marked improvements on beam walk with fewer foot slips in the course of repeated testing over days, indicating intact motor learning. We next performed a different test on motor skill using the complex running wheel; this task puts a greater demand on motor skill since only half of the complement of rungs are present on the wheel. The complex running wheel test measures motor skill distinct from the accelerated rotarod (62). We performed this test on male and female mice at ∼1.5 years of age. Mice were initially trained to run voluntarily on a normal wheel for 2 weeks, then replaced with a complex wheel missing more than half of the rungs in an alternate pattern. Due to high variability, we did not observe significant differences in daily maximal speed, distance traveled, and average velocity between WT and *Ctrp10* KO male mice on a complex running wheel (Fig. 7*C*). When first placed on a training wheel, we noted that the female KO mice had lower maximal running velocity, but not the distance traveled nor the average running velocity (Fig. 7*D*). When switched over to a complex wheel, *Ctrp10* KO female mice had markedly lower daily maximal speed, distance traveled, and average running velocity on a complex running wheel when compared to WT controls (Fig. 7*D*). Together, these results indicate that CTRP10 is required for optimal motor skill in negotiating an inclined beam walk and running on a complex wheel, with some sex differences noted.

### Loss of CTRP10 disrupts biological pathways in cerebellum and motor cortex

**Figure 8:**
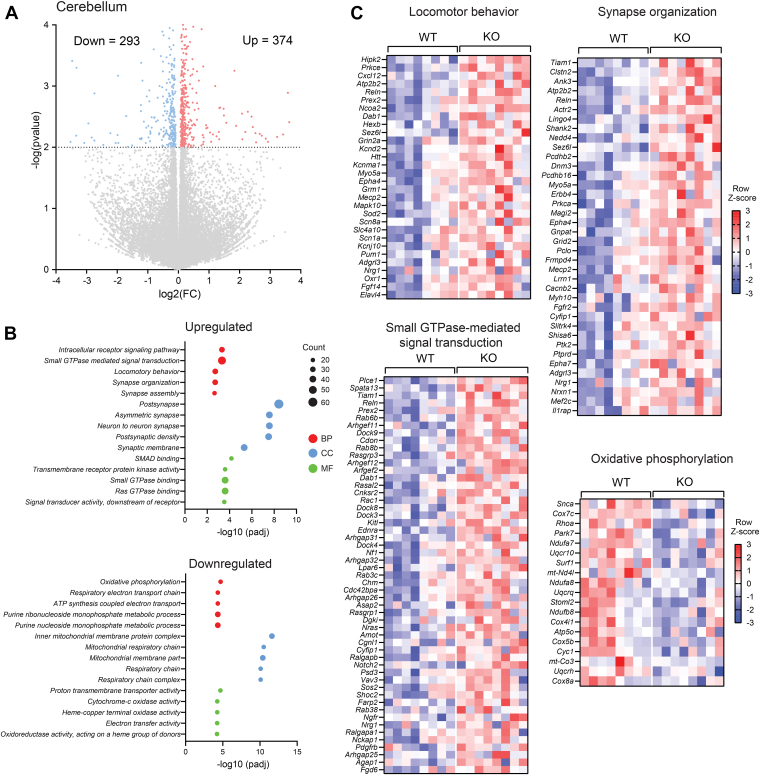
**Transcriptomic changes in the cerebellum of *Ctrp10* KO female mice**. *A*, volcano plot showing 371 upregulated and 291 downregulated genes in the cerebellum of *Ctrp10* KO females. *B*, gene ontology analysis showing biological process, cellular component, and molecular function that are altered in *Ctrp10* KO cerebellum. *C*, heatmaps of genes (based on gene ontology) involved in locomotor behavior, synapse organization, signaling, and mitochondrial oxidative phosphorylation. Sample size: WT = 8; KO = 8.

We performed transcriptomic analysis of the cerebellum and motor cortex—two brain regions critical for motor function— to gain molecular insights into mechanisms that could contribute to deficits in motor coordination and skill in *Ctrp10* KO mice. We focused this analysis on female mice since they exhibited a more pronounced phenotype as compared to males. We observed 374 upregulated and 293 downregulated genes in the cerebellum of *Ctrp10* KO female mice relative to WT controls (Fig. 8*A* and Tables S1 and S2). Expectedly, the most downregulated gene in the cerebellum of *Ctrp10* KO mice is *Ctrp10*/*C1ql2*, thus validating the absence of *Ctrp10* transcript in KO animals. Pathway enrichment analysis based on Gene Ontology was used to reveal biological process, cellular component, and molecular function up- or down-regulated in the cerebellum of *Ctrp10* KO mice. The top upregulated pathways include signaling, synapse assembly and organization, and locomotor behavior, and the top downregulated pathways are mostly associated with mitochondrial respiration and function (Fig. 8, *B* and *C*). Reduced expression of cerebellar OXPHOS genes likely contributes to a deficit in motor function in KO mice, as mitochondrial dysfunction is frequently associated with ataxia in numerous neurodegenerative diseases (63, 64, 65, 66).

**Figure 9:**
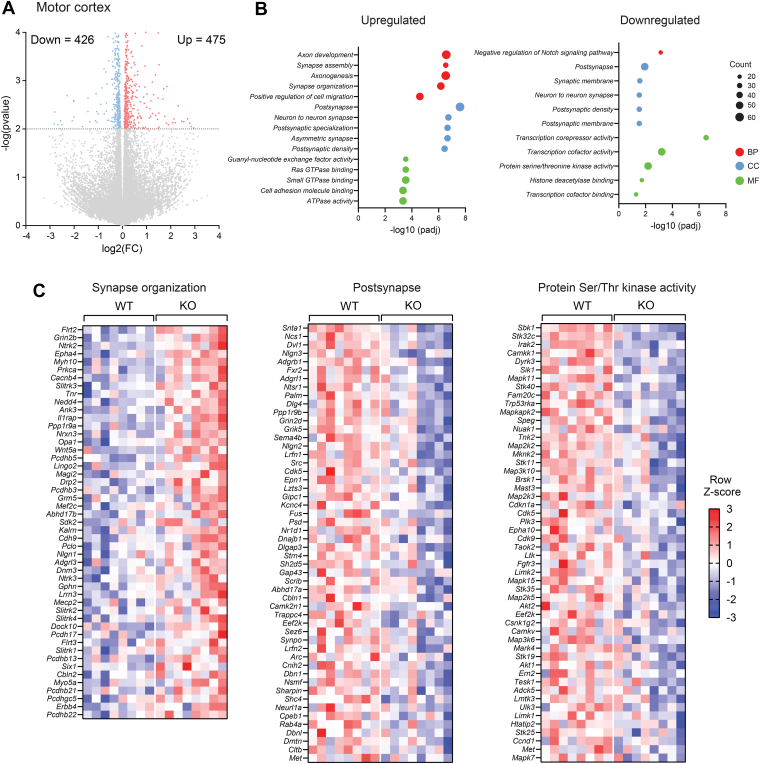
**Transcriptomic changes in the motor cortex of *Ctrp10* KO female mice**. *A*, volcano plot showing 476 upregulated and 426 downregulated genes in the motor cortex of *Ctrp10* KO females. *B*, gene ontology analysis showing biological process, cellular component, and molecular function that are altered in *Ctrp10* KO motor cortex. *C*, heatmaps of genes (based on gene ontology) involved in synapse organization, postsynapse, and Ser/Thr kinase activity. Sample size: WT = 8; KO = 8.

In the motor cortex, we observed 475 upregulated and 426 downregulated genes in *Ctrp10* KO female mice relative to WT controls (Fig. 9*A* and Tables S2 and S3). Again, as expected, the most down-regulated gene in motor cortex is *Ctrp10*/*C1ql2*. Gene Ontology analysis shows that the top upregulated pathways are related to synapse assembly and organization and cell signaling, and the top downregulated pathways are related to postsynaptic membrane and protein Ser/Thr kinase activity (Fig. 9, *B* and *C*). Several of the downregulated genes in cerebellum (*e*.*g*., *Sod1*, *Tuba4a*, and *Pycr2*) and motor cortex (*e*.*g*., *Dlg4*, *Nlgn2*, *Scyl1*, *Dctn1*, *Cbln1*, *Fxr2*, *Cdk5*) are known to play important roles in motor function, as mouse mutants lacking these genes have impaired motor coordination and function (67, 68, 69, 70, 71, 72, 73, 74, 75, 76). Conversely, some of the upregulated genes in cerebellum (*e*.*g*., *Reln*, *Scn1a*) and motor cortex (*e*.*g*., *Chd8*, *Scn1a*) are known to play a negative role in motor function, as mutant mice lacking one or both copy of these genes have improved motor coordination (77, 78, 79); thus, increased expression of these genes may adversely affect motor function in *Ctrp10* KO mice. Together, these data highlight gene expression and pathways changes in the cerebellum and motor cortex that contribute to motor function deficits seen in *Ctrp10* KO mice.

### CTRP10 deficiency affects shared and distinct genes in cerebellum and motor cortex

**Figure 10:**
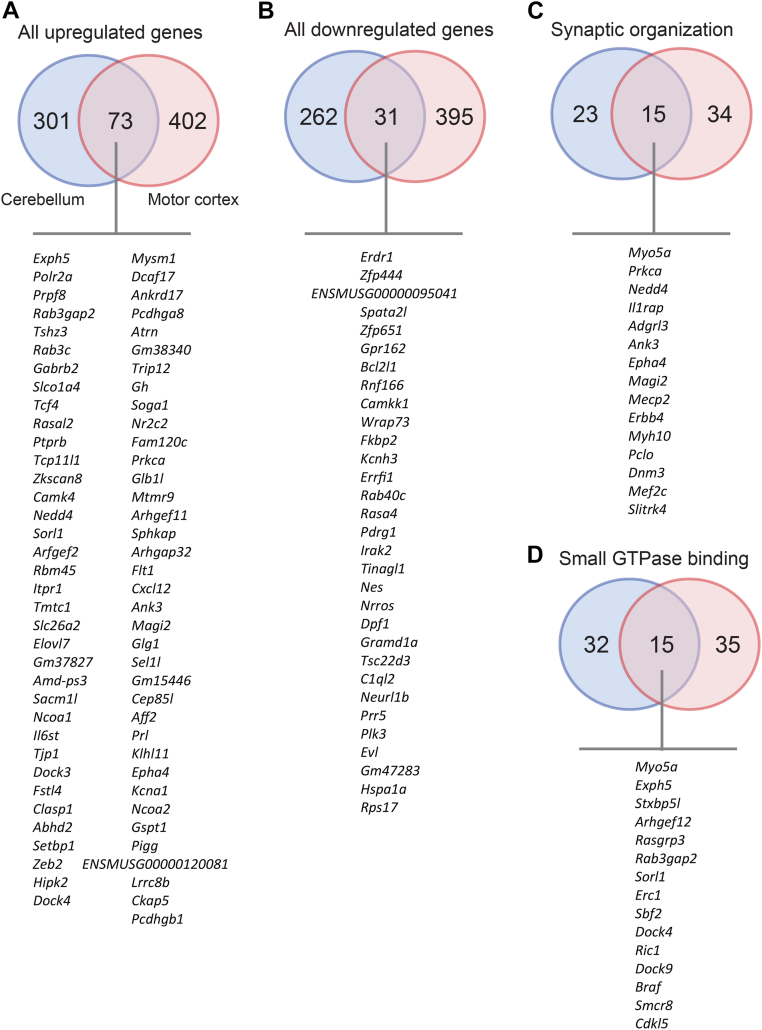
**Shared differentially expressed genes in the cerebellum and motor cortex of *Ctrp10* KO female mice**. *A*, a total of 73 upregulated genes are shared between the cerebellum and motor cortex. *B*, a total of 31 downregulated genes are shared between the cerebellum and motor cortex. *C*, a total of 15 upregulated genes involved in synaptic organization is shared between the cerebellum and motor cortex. *D*, a total of 15 upregulated genes involved in small GTPase binding are shared between the cerebellum and motor cortex.

We performed overlap analysis to highlight the shared differentially expressed genes between cerebellum and motor cortex. Among the upregulated and downregulated genes, 73 and 31 are shared between cerebellum and motor cortex, respectively (Fig. 10, *A* and *B*). Among the upregulated genes involved in synaptic organization (based on Gene Ontology), 15 are shared between cerebellum and motor cortex (Fig. 10*C*). And among the upregulated genes involved in small GTPase binding, 15 are shared between cerebellum and motor cortex (Fig. 10*D*). Thus, loss of CTRP10 dysregulates the expression of shared and distinct genes across cerebellum and motor cortex. While some of the biological processes and molecular functions affected by CTRP10 deficiency are similar between cerebellum and motor cortex, the gene set involved only partially overlaps. These data suggest common and distinct transcriptional changes across cerebellum and motor cortex in response to *Ctrp10* deletion, the combination of which likely underpins the motor phenotype observed in KO animals.

### Mitochondrial respiration is reduced in the motor cortex of *Ctrp10* KO mice

**Figure 11:**
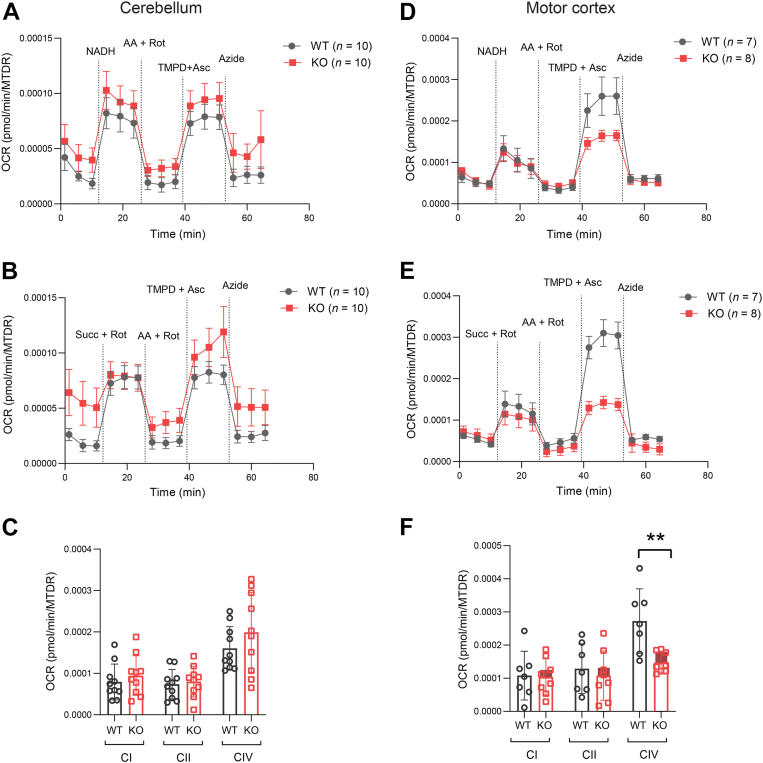
**Reduced mitochondrial respiration in the motor cortex of *Ctrp10* KO female mice**. Mitochondrial respiration in the cerebellum (*A*–*C*) and motor cortex (*D*–*F*) of WT and KO female mice. The following *panels* (from *top to bottom*) are shown: *PanelA* and *D*: maximal oxygen consumption rate of mitochondrial complex I and CIV. *PanelB* and *E*: maximal oxygen consumption rate of mitochondrial complex complex II and CIV. *PanelC* and *F*: Quantification of maximal oxygen consumption rate of mitochondrial complex complex I, complex II, and CIV. All oxygen consumption data are normalized to mitochondrial content. Sample size: WT = 10; KO = 10. All data are presented as mean ± S.D. ∗∗*p* < 0.01 (unpaired two-tailed student *t* test). CIV, complex IV.

Since genes related to mitochondrial respiration and function are downregulated in the cerebellum of *Ctrp10* KO female mice, we performed high-resolution respirometry analysis to assess mitochondrial function. Using different substrate as electron donor, this assay allows the determination of mitochondrial activity through complex I (CI), complex II (CII), and complex IV (CIV) separately. We used NADH as electron donor for CI, succinate as electron donor for CII, and TMPD as electron donor for CIV. Contrary to expectation, in the cerebellum, mitochondrial respiration through CI, CII, or CIV was not significantly different between genotypes in female mice (Fig. 11, *A*–*C*). In striking contrast, respiration at CIV (cytochrome c oxidase)—the last protein complex and final electron acceptor in the electron transport chain—was markedly lower in the motor cortex of *Ctrp10* KO female mice (Fig. 11, *D*–*F*). These data suggest that a functional deficit in mitochondrial respiration in the motor cortex of *Ctrp10* KO mice may partly contribute to impaired motor function.

## Discussion

Multiple recent studies, largely based on association, have hinted a potential central role for CTRP10/C1QL2 in regulating memory (43, 46), cognition (46), mood (45), sensorimotor gating (45), and fine motor skill (47). The contribution of CTRP10 to these CNS functions, however, has not been directly demonstrated. In this study, we set out to address whether CTRP10 is required for CNS control of mouse behaviors using a genetic loss-of-function mouse model. We show that CTRP10 is not essential for general exploratory behavior, anxiety-like responses, spatial working and recognition memory, and sensorimotor gating. However, it is required for optimal motor function. Mice lacking CTRP10 have striking deficits in gross motor coordination and fine motor skill. The motor phenotypes are associated with major transcriptomic and pathway changes in the cerebellum and motor cortex, as well as reduced mitochondrial respiration in the motor cortex.

In humans, sex differences in motor performance in both healthy and disease states are well documented, with females generally having poorer motor function compared to males (80, 81, 82, 83). Interestingly, the phenotypic outcomes due to CTRP10 loss are also sex-dependent. Female *Ctrp10* KO mice, but not males, exhibit pronounced deficits in motor coordination as determined by the standard rotarod test. This is not entirely unexpected, as sexual dimorphism in neuromotor phenotypes has been reported in other genetic models, potentially reflecting sex hormone modulation or differential compensatory capacity (84, 85). Deficits extended to fine motor skills, evidenced by increased slips on the inclined beam walk and diminished performance on the complex running wheel. Notably, these impairments were most pronounced in females. Importantly, grip strength remains intact in KO mice, thus ruling out intrinsic defects in skeletal muscle that can affect motor performance. In all our motor function tests, motor learning—as indicated by improved performance with successive trials—is preserved in CTRP10-deficient mice. This suggests that the primary deficit is in motor execution or output, rather than in plasticity mechanisms themselves. Our results are consistent with studies indicating that cerebellar lesions can selectively impair coordination and skill without overtly affecting motor learning (86). There is one caveat regarding our findings: rotarod performance can be influenced by body weight. Since the female KO mice exhibited higher body weights (41), we cannot entirely rule out weight as a potential confounding factor in our results.

Our transcriptomic analyses revealed broad gene expression changes in cerebellum and motor cortex of *Ctrp10* KO females, regions integral to motor output. A subset of differentially expressed genes—73 upregulated and 31 downregulated genes—were shared between cerebellum and motor cortex, indicating shared and region-specific molecular changes induced by CTRP10 loss. Upregulation of genes linked to synapse assembly/organization and small GTPase-mediated signaling, alongside downregulation of genes related to post-synapse, mitochondrial respiration, and Ser/Thr kinase activity in cerebellum and/or motor cortex likely contributed to the motor phenotypes seen in our KO animals. Key genes such as *Sod1*, *Tuba4a*, *Pycr2*, *Dlg4*, *Nlgn2*, *Scyl1*, *Dctn1*, *Cbln1*, *Fxr2*, and *Cdk5*—all critical for motor function—were among those downregulated, consistent with prior reports that their disruption impairs motor coordination and function (67, 68, 69, 70, 71, 72, 73, 74, 75, 76). Conversely, increased expression of *Reln*, *Scn1a*, and *Chd8*, which can negatively regulate motor function (77, 78, 79), further supports the link between transcriptomic changes and altered motor performance.

Mitochondrial dysfunction is a common feature of many neurodegenerative diseases and ataxias (66, 87, 88). We know that the cortex and cerebellum have high mitochondrial respiration (89). In our study, OXPHOS transcript downregulation in the cerebellum did not translate to detectable deficits in cerebellar mitochondrial respiration. In contrast, although OXPHOS transcripts were not significantly altered in the motor cortex, we observed a marked impairment in mitochondrial respiration in KO females. This suggests that CTRP10 loss negatively impacts mitochondrial function in a brain region-specific manner and that the motor cortex is more sensitive to CTRP10 loss. This selective vulnerability of the cortex, and not the cerebellum, to mitochondrial dysfunction is reminiscent of anatomical selectivity seen in other mouse models of neurodegenerative diseases (90, 91). Reduced mitochondrial respiration will affect the energetics of neurotransmission and synaptic vesicle refilling and recycling (92, 93). Thus, our results support a deficit in mitochondrial function as a plausible contributing factor for impaired motor function in *Ctrp10* KO animals.

We attributed the motor phenotype of *Ctrp10* KO mice to major transcriptomic changes and reduced mitochondrial respiration in the motor cortex and/or cerebellum; however, the mechanism linking these molecular and functional changes to CTRP10 loss remains to be clarified. Previous studies have indicated a transsynaptic role for CTRP10/C1QL2 at the mossy fiber-CA3 synapses in the hippocampus (42, 43). It has been shown that CTRP10 interacts with multiple proteins at the presynaptic and postsynaptic terminals, including Nrx3 and kainate-type GluK2 and GluK4. Additionally, CTRP10 has also been shown to bind to the adhesion GPCR, Bai3 (94). Future studies are warranted to determine whether CTRP10 plays a similar transsynaptic role in the motor cortex and cerebellum, and whether CTRP10 loss disrupts synaptic integrity leading to altered neuronal signaling and gene expression. This possibility is suggested by our observation that the major biological pathways altered in *Ctrp10* KO mouse cerebellum and motor cortex are those related to synapse organization and signaling.

We wish to highlight several limitations of our study. Our findings are based on the use of a constitutive whole-body KO mouse model. It is known that CTRP10/C1QL2 is expressed during development in humans and mice (95, 96). Although our data suggest that CTRP10 is dispensable for spatial working and recognition memory, and sensorimotor gating, we cannot rule out potential compensation during development; this can be addressed in future studies using conditional KO models to selectively delete *Ctrp10* gene in the brain of adult mice. Both neurons and oligodendrocyte progenitor cells robustly expressed *Ctrp10* transcript (51, 52). In one study using the Drop-seq method, neurons in the hippocampus and substantia nigra expressed the highest levels *Ctrp10* (52). Thus, we expect conditional models to selectively delete *Ctrp10* in different cell populations of the brain will help clarify the cell-type requirement of CTRP10 in controlling motor function and possibly other behavioral outputs. Given the documented role of CTRP10 in the hippocampus (43), additional behavioral tests, such as Morris water maze and contextual fear conditioning, would also be informative and allow us to determine whether hippocampal-dependent learning and memory is affected in our *Ctrp10* KO mice. Age can be a confounding factor in our mouse behavioral tests. Specifically, the prepulse inhibition test was performed on older females at 39 weeks of age. Since C57BL/6 mice are known to experience age-associated hearing loss (97), we cannot definitively rule out whether CTRP10 loss affects sensorimotor gating. Additionally, while we did not observe sex differences in the open field, elevated maze, Y-maze, or novel object recognition tests, these tests were conducted on KO male and female mice at varying ages. Consequently, potential sex differences in these behaviors may have been masked by age differences. This issue can be clarified in future studies by using younger female mice.

In summary, we provided genetic evidence to show that CTRP10 is required for optimal gross and fine motor function. Given the sparse functional data concerning CTRP10, our study helps fill a major knowledge gap and establishes essential baseline information needed to inform future studies aim at dissecting the mechanistic link of CTRP10’s action in the CNS and its associated behavioral outputs.

## Experimental procedures

### Mouse model

The *Ctrp10/C1ql2*-KO mice (C57BL/6NCrl-C1ql2em1(IMPC)Mbp/Mmucd; stock number 050587-UCD) were generated using the CRISPR-cas9 method at UC Davis, and detailed genotyping protocol was previously described (41). All mice were generated by intercrossing *Ctrp10* heterozygous (+/−) mice. *Ctrp10* KO (−/−) and WT (+/+) littermate controls were housed in polycarbonate cages on a 12-h light–dark photocycle with ad libitum access to water and food. The male and female mice used in the study were from different breeding cohorts. Consequently, there was a difference in age between the two groups. Mice were fed a standard chow (Lab Diet; 5V75). All mouse protocols were approved by the Institutional Animal Care and Use Committee of the Johns Hopkins University School of Medicine. All animal experiments were conducted in accordance with the National Institute of Health guidelines and followed the standards established by the Animal Welfare Acts.

### Open field

All behavioral tests described in this study were conducted by experimenters blinded to genotypes, and all tests were performed during the active (dark cycle) phase of the mouse circadian cycle, as we have previously described (98, 99). Locomotor activity was assessed over 30 min in a 40 x 40 cm activity chamber with infrared beams (San Diego Instruments Inc.). Horizontal activity, as well as time spent in the center or periphery of the chamber was automatically recorded.

### Elevated plus maze

Anxiety related behavior was evaluated using the elevated plus maze test. Mice were placed in the center of a 54 cm high maze consisting of two open and two closed (30.5 cm long) arms (San Diego Instruments Inc.) for 5 min. Distance traveled and time spent in the open and closed arms was automatically recorded using Anymaze Tracking Software (Stoelting, Co., https://www.any-maze.com).

### Y-maze spontaneous alternation

Working memory was assessed in the Y-maze spontaneous alternation task. Mice were placed at the end of one arm of a Y-maze consisting of three 38 cm long arms (San Diego Instruments Inc.) and allowed to explore the maze for 5 minutes. Distance traveled and entries into the arms were automatically recorded using Anymaze Tracking Software (Stoelting, Co.). The percent alternation was calculated using the equation % Alternation = (correct alternations/(total arm entries – 2))∗100.

### Novel object recognition test

The novel object recognition test was used to evaluate short term memory. Briefly, mice were allowed to habituate to an empty 20 cm × 20 cm box for 10 min. Twenty-four hours following habituation, mice were placed in the box with two identical objects for a 10-min training period. 30 minutes following the training period, one object was replaced with a novel object, and the mouse was allowed to explore the objects for 5 minutes. Time spent sniffing the novel and familiar objects was measured using CleverSys Topscan tracking software (CleverSys Inc., https://cleversysinc.com/). The objects used for the novel object recognition test were square blocks and spheres from Stoelting (Stoelting,). Our pilot data shows no difference in the percent time spent with the sphere *versus* percent time spent with the squares. Despite a lack of clear object preference, additional steps were taken during testing to account for a potential object preference by presenting half of each group with the square as the novel object and half of each group with the sphere as the novel object. Other factors were also taken into consideration and counterbalanced among groups, including the novel object side and novel object box position.

### Prepulse inhibition of acoustic startle response

Sensorimotor gating was evaluated by prepulse inhibition (PPI) as described (100). The experimental session consisted of a 5-min acclimatization period to a 70-dB background noise (continuous throughout the session), followed by the presentation of 10 40-ms 120-dB white noise stimuli at a 20-s inter-stimulus interval (the habituation session). Upon the completion of the habituation session, each mouse was left in the enclosure for 5 min without presentations of any startle stimuli. Immediately after, the pre-pulse inhibition (PPI) session began. During each PPI session, a mouse was exposed to the following types of trials: pulse-alone trial (a 120-dB, 100-ms, broadband burst); the omission of stimuli (no-stimulus trial); and five pre-pulse–pulse combinations (pre-pulse–pulse trials) consisting of a 20-ms broadband burst used as a pre-pulse and presented 80 ms before the pulse using one of the five pre-pulse intensities: 74; 78; 82; 86 and 90 dB. Each session consisted of six presentations of each type of the trial presented in a pseudorandom order. PPI was assessed as the percentage scores of PPI (%PPI): 100 × (mean startle amplitude on pulse-alone trials – mean startle amplitude on prepulse–pulse trials/mean startle amplitude on pulse-alone trials) for each animal separately. The percentage of PPI for each animal was used as the dependent variable in statistical analysis.

### Grip strength test

Grip strength was measured using a Bioseb grip strength meter (BIO-GS3, Bioseb) as we have previously described (101). In brief, mice were held in front of a grid until the forepaws grabbed the grid. Mice were then pulled horizontally by the tail until they lost grip of the grid. Their peak pull force was recorded in each of three trials and averaged.

### Motor coordination and learning test

Motor coordination and learning was evaluated using the accelerating rotarod test as we have previously described (99). Briefly, mice were placed on the rotarod (Columbus Instruments) with a starting speed of 4 RPM, with an acceleration of 7.2 RPM/minute. The time at which each mouse dropped from the rotating rod was recorded. Each mouse was given three trials per day with a 2-min inter-trial interval, for 3 days.

### Beam walk test

The inclined beam-walking test was performed as we have previously described (102). In brief, an elevated 80 cm in length wooden beams was placed at a 30° angle. A dark box with bedding was at the end of the incline and served as a target for the mouse to reach. A blinded experimenter observing and recording from above assessed mouse performance by documenting the number of foot slips (either hind legs or front legs) and the time to traverse the beam.

### Complex running wheel test

The complex running wheel test was performed as we have previously described (102). In brief, mice were individually housed in a modified cage equipped with a running wheel attached to an optical sensor to detect the number of wheel revolutions per time interval (minute). During the first 2 weeks, a training wheel with all 38 rungs was present; this allows for normalization of running behavior. In the third week, the regular training wheel was replaced with a complex wheel of the same diameter with 22 rungs missing in an alternate pattern. Using Activity Wheel Monitoring Software (Lafayette Instruments, https://lafayettelifescience.com/), wheel revolutions were recorded each day and exported to a Microsoft Excel file in which daily total distance traveled and maximum daily velocity were calculated. To effectively analyze the data generated from the complex running wheel, we developed an analysis pipeline using shorthand macros in Microsoft Excel. All mice showed spontaneous running behavior and no mice were excluded from this study.

### Mitochondrial respirometry

Respirometry was conducted on frozen tissue samples to assay for mitochondrial activity as described previously (89, 103). Briefly, mouse motor cortex and cerebellum were dissected, snap frozen in liquid nitrogen, and stored at −80 °C for later analysis. Samples were thawed in MAS buffer (70 mM sucrose, 220 mM mannitol, 5 mM KH_2_PO_4_, 5 mM MgCl_2_, 1 mM EGTA, 2 mM Hepes pH 7.4), finely minced with scissors, and then homogenized with a glass Dounce homogenizer. The resulting homogenate was spun at 1000 *g* for 10 min at 4 °C. The supernatant was collected and immediately used for protein quantification by BCA assay (Thermo Fisher Scientific, 23,225). Each well of the Seahorse microplate was loaded with 6 μg of homogenate protein. Each biological replicate consisted of three technical replicates. Samples were treated separately with NADH (a CI substrate, 1 mM) or Succinate (a complex II substrate, 5 mM) in the presence of rotenone (a CI inhibitor, 2 μM), then with the inhibitors rotenone (2 μM) and Antimycin A (4 μM), followed by TMPD (0.45 mM) and Ascorbate (1 mM) to activate complex IV, and finally treated with Azide (40 mM) to assess non-mitochondrial respiration. All mitochondrial respiration data were normalized to mitochondrial content, quantified using MitoTracker Deep Red (MTDR, Thermo Fisher Scientific, M22426) as described (89, 103). Briefly, lysates were incubated with MTDR (1 μM) for 10 min at 37 °C, then centrifuged at 2000 *g* for 5 min at 4 °C. The supernatant was carefully removed and replaced with 1x MAS solution and fluorescence was read with excitation and emission wavelengths of 625 nm and 670 nm, respectively. To minimize non-specific background signal contribution, control wells were loaded with MTDR and 1x MAS and subtracted from all sample values.

### RNA-sequencing and bioinformatics analysis

To minimize biases in transcript sampling, total RNA was isolated from one hemispheric side of the entire cerebellum and motor cortex. Bulk RNA sequencing of female WT (*n* = 8) and *Ctrp10*-KO (*n* = 8) mouse cerebellum and motor cortex were performed by Novogene (Sacramento) on a NovaSeq X Plus platform and pair-end reads (2 x 150 bp) were generated, with 6 gigabyte raw data per sample. Sequencing data was analyzed using the standard Novogene Analysis Pipeline. Sequencing reads were aligned to *Mus musculus* reference genome (GRCm39/mm39). Data analysis was performed using a combination of programs: Fastp, Hisat2, and FeatureCounts. Differential expressions were determined through DESeq2. The *p*-values of DEGs were adjusted for false discovery rate using the Benjamini and Hochberg’s approach. Gene ontology, Kyoto Encyclopedia of Genes and Genomes, and Reactome enrichment were implemented by ClusterProfiler. All volcano plots and heat maps were generated in GraphPad Prism 10 software (https://www.graphpad.com/). All statistics were performed on Log transformed data. All heat maps were generated from z-score transformed data. The z-score for each gene is defined as: (individual expression value - the average for all samples)/(standard deviation for all samples). High-throughput sequencing data from this study have been submitted to the NCBI Sequence Read Archive under the accession number # PRJNA1168975.

### Statistical analyses

Statistical analysis was performed with Prism 10 software (GraphPad Software). Data were analyzed with unpaired two-tailed Student’s *t*-tests or by repeated measures ANOVA. *p* < 0.05 was considered statistically significant. All data are presented as mean ± SD (standard deviation).

## Data availability

High-throughput sequencing data from this study have been submitted to the NCBI Sequence Read Archive under accession number PRJNA1168975.

## Supporting information

This article contains supporting information.

## Conflic of interest

The authors declare that they have no conflicts of interest with the contents of this article.
