# Supplementary material for: CTRP10 is required for optimal motor function
Source: J Biol Chem. 2026 Mar 31;302(5):111420. doi: 10.1016/j.jbc.2026.111420 (PMC13126028; doi:10.1016/j.jbc.2026.111420)
Supplement: Supplementary Material 5 [file mmc5.docx]

**Supporting Information**

**Supplementary Table S1.** All the differentially expressed genes (DEGs) that are upregulated in the cerebellum of *Ctrp10* KO female mice relative to wild-type (WT) controls.

**Supplementary Table S2.** All the differentially expressed genes (DEGs) that are downregulated in the cerebellum of *Ctrp10* KO female mice relative to wild-type (WT) controls.

**Supplementary Table S3.** All the differentially expressed genes (DEGs) that are upregulated in the motor cortex of *Ctrp10* KO female mice relative to wild-type (WT) controls.

**Supplementary Table S4.** All the differentially expressed genes (DEGs) that are downregulated in the motor cortex of *Ctrp10* KO female mice relative to wild-type (WT) controls.
